# UCHL1 regulates oxidative activity in skeletal muscle

**DOI:** 10.1371/journal.pone.0241716

**Published:** 2020-11-02

**Authors:** Hongbo Gao, Ryan Antony, Rekha Srinivasan, Penglong Wu, Xuejun Wang, Yifan Li

**Affiliations:** Division of Basic Biomedical Sciences, Sanford School of Medicine, University of South Dakota, Vermillion, SD, United States of America; University of Debrecen, HUNGARY

## Abstract

Ubiquitin C-terminal Hydrolase L1 (UCHL1) is a deubiquitinating enzyme that was originally identified in neurons. Our recent study showed that UCHL1 was expressed in C2C12 myoblast cells and mouse skeletal muscle. Here we report that in mouse skeletal muscle, UCHL1 is primarily expressed in oxidative muscle fibers. Skeletal muscle specific gene knockout (smKO) of UCHL1 in mice reduced oxidative activity in skeletal muscle measured by SDH staining. The *in situ* muscle contraction test revealed that gastrocnemius muscle from UCHL1 smKO mice was more prone to fatigue in response to the repetitive stimulation. This data suggests that UCHL1 plays a role in maintenance of muscle oxidative metabolism. Moreover, UCHL1 smKO caused a significant reduction in key proteins that are involved in mitochondrial oxidative phosphorylation in soleus muscles, suggesting that UCHL1 may be involved in regulation of mitochondrial content and function. Immunostaining showed the co-localization of UCHL1 and mitochondrial marker VDAC in skeletal muscle. Mitochondrial fractionation assay revealed that, although UCHL1 was primarily present in the cytosolic fraction, a low level of UCHL1 protein was present in mitochondrial fraction. The level of phosphorylation of AMPKα, a master regulator of mitochondrial biogenesis, were unchanged in UCHL1 smKO muscle. On the other hand, immunoprecipitation from soleus muscle sample indicated the interaction between UCHL1 and HSP60, a chaperon protein that is involved in mitochondrial protein transport. There was a trend of downregulation of HSP60 in UCHL1 smKO muscle. Overall, our data suggests UCHL1 is a novel regulator of mitochondrial function and oxidative activity in skeletal muscle.

## Introduction

Skeletal muscle is the most abundant tissue in the body, accounting for over 40% of total body mass and playing a critical role in metabolism and energy homeostasis in physiological [1, 2] and pathophysiological conditions [3]. Skeletal muscles are heterogeneous tissues that are composed of diverse myofibers distinguished by special types of myosin heavy chain (MyHC), function, and metabolism. Type I and type IIa muscle fibers generate ATP by oxidation of fatty acid or glucose, whereas glycolytic type IIb muscle fibers generate ATP through glycolysis [4]. Alteration of skeletal muscle metabolism and muscle fiber composition are commonly observed in various health conditions including type II diabetes [5], heart failure [6], chronic obstructive pulmonary diseases [7], and aging [8–10]. The mechanisms underlying the metabolic plasticity within muscle remain to be fully understood.

Ubiquitin C-terminal hydrolase L1 (UCHL1) was originally identified as a neuronal protein and later defined as a deubiquitinating enzyme [11]. Despite substantial investigations over the decades, the downstream substrates and functional importance of UCHL1 are still poorly understood [12]. The expression and function of UCHL1 in skeletal muscle was essentially unknown until recently. It was recently reported that UCHL1 was expressed in mouse skeletal muscle in disease conditions [13, 14]. We recently showed that UCHL1 was expressed in C2C12 mouse myoblast cells and was involved in the regulation of cell proliferation and differentiation [15]. To study the function of UCHL1 in skeletal muscle, we have recently developed a mouse model with skeletal muscle specific knockout (smKO) of UCHL1. Using this novel model, here we report that UCHL1 is involved in skeletal muscle oxidative activity.

## Methods

### Animals

The animal study in this manuscript was approved by University of South Dakota IACUC. The protocol No. is 1-03-19-22D. In some of experiments in this study, mice were anesthetized using isoflurane inhalant to effect. For euthanasia, under deep anesthesia with isoflurane (4%-5%), tissues of interest were collected and then animals sacrificed by cervical dislocation and thoracotomy. To understand the functional role of UCHL1 in skeletal muscle oxidative function, we generated a mouse line with skeletal muscle specific knockout (smKO) of UCHL1. The mouse strain “UCHL1 HEPD0603 _7_h04” generated from EUCOMM/KOMP-CSD ES cells [16–18] was provided by Medical Research Council (MRC)-Harwell, UK, on behalf of the European Mouse Mutant Archive (EMMA) and the International Mouse Phenotyping Consortium (IMPC, www.mousephonogype.org). By crossing this strain with a mouse expressing Flp recombinase, the targeting cassette (LacZ and Neo genes) were removed (Fig 2A). Further breeding with wild type (WT) mice separated the Flp transgene out, the resulting strain carrying the exon 2 floxed *UCHL1* gene and free of Flp was then crossed to a mouse expressing Cre recombinase driven by the myosin light chain 1 promoter (Myl1^tm1(cre)sjb^/J, Jackson Lab, stock # 024713) [19] to generate a mouse strain with skeletal muscle specific UCHL1 knockout (smKO). Genotyping was conducted using PCR with appropriate primers for floxed UCHL1 and Cre.

### Tissue collection

As described previously [20], mice were anesthetized with isoflurane. Hindlimb muscles were carefully exposed by removing skin. Soleus, extensor digitorum longus (EDL), and gastrocnemius were collected and frozen on dry ice for Western blot assay. Soleus, EDL, and gastrocnemius are primarily composed of slow oxidative fibers, fast glycolytic fibers, and mixed muscle fibers, respectively. For tissue staining, gastrocnemius muscles were isolated and snap frozen in pre-chilled 2-methylbutane for ~20 seconds [21] and transferred to -70°C until sectioning. Mid-belly region of muscles was sectioned into 10 μm thickness and adhered on the slides. The slides were stored at -70°C for future staining.

### Succinate dehydrogenase (SDH) stain

The methods was based on published work [22] with minor modification. In brief, SDH working solution was freshly prepared by dissolving 8.4 mg 1-methoxyphenzine methosulphate (mPMS), 30.7 mg Nitroblue tetrazolium (NBT), and disodium succinate hexahydrate (- final concentration 50 mM) in 25 ml phosphate buffer (pH 7.6). Slides were incubated in the SDH working solution for 10 mins and rinsed with distilled water. Slides were mounted with DPX mounting media (Sigma-Aldrich) and dried in the dark at room temperature.

### Tissue immunostaining

As described previously [23], the slides with frozen muscle sections were thawed and air dried for 20 min at room temperature. The sections were incubated in TBST for 5 min and the fixed with 4% paraformaldehyde (PFA) for 10 min. After rinse with TBST, sections were blocked with 3% bovine serum albumin (BSA) and 10% AffiniPure Goat Anti-Mouse IgG + IgM Fab (H+L) (Jackson ImmunoResearch, 115-005-044) TBST solution for 1 h at room temperature. Then sections were incubated with primary antibodies diluted in 3% BSA and 5% donkey serum TBST solution overnight at 4°C. Rabbit anti UCHL1 (abcam 108986) and mouse anti type I and type IIa oxidative MyHC (Developmental Studies Hybridoma Bank, BF-32) were used. Following 3 washes with TBST, sections were incubated with appropriate secondary antibodies conjugated with fluorescent dye Alexa 594 or Alexa 488 diluted in 3% BSA TBST (1:400) for 1 hour at room temperature. Sections were then incubated with Hoechst in TBST (1:15 000) for 5 min, followed by 2 washes with TBST. The sections were mounted with coverslips using Fluomount-G solution (Southern Biotech, 0100–01). Immunostaining images was taken using a confocal microscope (Olympus Fluoview 500, Center Valley, PA).

### Total protein extraction and Western blot analysis

As described previously [20, 23], muscle tissues were homogenized in 1X RIPA buffer containing freshly added 1% protease inhibitor cocktail (Santa Cruz, Dallas, TX) and 1% phosphatase inhibitor (Research Product International, Mount Prospect, IL). Protein concentration of samples was determined by a standard BCA assay and normalized.

For mitochondrial fractionation, approximately 50 mg of muscle tissue was washed with PBS and homogenized on ice. Mitochondrial and cytosolic fractions of the homogenates were isolated using a mitochondria isolation kit (Thermo Scientific, 89801). The resultant fractions were subjected to Western blot.

Western blot was performed as described previously [20, 23, 24]. Briefly, samples of 15–30 μg of total proteins were subject to electrophoresis in 9–15% gradient gels at 100 V for 2 hours. Proteins were transferred onto nitrocellulose membranes at 300 mA using a Trans-blot apparatus (Bio-Rad, Hercules, CA). Blocked with 3% non-fat milk in PBST solution for 1 hour, membranes were incubated with primary antibodies overnight at 4°C. Following primary antibodies were used: anti UCHL1 (abcam 108986), OXPHOS antibody cocktail (abcam 110413), SDHA (abcam, AMPKα (Cell Signaling technologies, 2793), phospho-AMPKα (Cell Signaling Technologies, 2535), CPT1 (ProteinTech, 15184), HA (ProteinTech, 51064), VDAC1 (Biolegend, N152B/23), HSP60 (Biolend, P83G8), and GAPDH (sc-47724). After 3 washes with PBST, membranes were incubated with the appropriate secondary antibodies conjugated with Alexa-680 or 800 (Invitrogen) for 1 hour at room temperature followed by 3 washes with PBST. The protein bands on the membrane were detected using LI-COR scanner (LI-COR Biosciences, Lincoln, NE). The band densities of proteins were measure using NIH ImageJ software and normalized against the band density of GAPDH and then the ratio of UCHL1 KO versus WT was determined and compared.

### Mitochondrial isolation and complex II/III activity assay

Freshly isolated soleus muscles from WT or UCHL1 mKO mice were homogenized. Mitochondria were isolated using a commercial kit (ThermoFisher). The activities of mitochondrial electron transport complex II/III were measured using a commercial kit (Cayman Chemical) following the manufacturer’s instruction.

### *In situ* muscle contraction

Mice were anesthetized using isoflurane at 3% as an initial dose and 1.5% for maintenance. The skin of the right hind limb was removed and the gastrocnemius-plantaris-soleus muscle complex (GPSC) or tibialis anterior (AT) were isolated from surrounding tissues with nerve and blood supply intact; the distal tendon was sutured, cut off, and connected to a force transducer (MLT1030/D, ADInstruments Inc.). A bipolar electrode was located at the proximal end of gastrocnemius to avoid neuro-muscular junction dependency and a pulse with 8 V in amplitude and 10 ms in width was used, which induces a whole muscle contraction. The muscle performance and fatigue were tested using following protocols: (1) Contractile force at various preload: A single contraction was induced at various preloads ranged from 4 to 18 g with a 2 g increment; (2) frequency-peak force curve: a tetanic contraction was induced with various frequencies from 20 to 120 Hz with a 20-Hz increment. The peak contraction force was plotted against frequency; (3) fatigue: repetitive single contractions were induced at a rate of 2 Hz for 4 minutes and the decline of contractile force over time was used to measure muscle fatigue.

### Cell culture, UCHL1 overexpression, and immunoprecipitation

As previously described [15], C2C12 myoblasts were cultured in growing medium (GM) which was Dulbecco’s Modified Eagle’s Medium (DMEM, ATCC) supplemented with 10% fetal bovine serum (FBS, GE HyClone, Logan, UT) and 1% penicillin-streptomycin. Growing to 80–90% confluence, cells were infected with adenovirus expressing mouse UCHL1 tagged with HA (Ad.UCHL1-HA) in differentiating medium (DM) that was DMEM supplemented with 2% horse serum (GE HyClone) and 1% penicillin-streptomycin. After 24 hours of infection, old media were replaced by fresh DM and cells were further differentiated for 2 more days and then were harvested and lysed in 500 μL of ice-cold lysis buffer (20 mM Tris HCl, pH 8.0, 137 mM NaCl, 2 mM EDTA, 1% NP-40, and protease inhibitor cocktail added freshly). After, cell lysate supernatant was incubated with 3 μg of a mouse anti HA antibody with rotation for 2 hours at 4°C. Then pre-washed 50 μl of 50% slurry of protein G-agarose beads was added and the incubation was continued overnight at 4°C. Protein G-agarose beads were pulled down by centrifugation at 150 X g for 5 mins and were washed three times with wash buffer (10 mM Tris [pH 7.5], 150 mM NaCl, 1 mM EDTA, 1 mM EGTA, and 1% Triton X-100), The beads were boiled in lysis buffer containing beta mercaptoethanol (BME) loading buffer for 5 mins to elute the proteins. The protein samples were analyzed using Western blot.

### Data analysis

Data calculation, graphing and descriptive statistics were performed using Microsoft Excel Data Analysis Package or GraphPad Prism 7.03. For Western blot quantifications, a protein band density was normalized by GAPDH band as a loading control. The mean value of the WT group was calculated, followed by determining a ratio between each individual sample and that of the mean WT value. Data was presented as mean ± standard deviation (SD). The quantifications of tissue staining were conducted with three mice per group, three -four sections per mouse, and four to five randomly fields per section. Total and positively stained fibers in each field were counted and the percentage of positive versus total fibers in the chosen areas was determined. Statistical significance of WT and KO group was compared by one-factor analysis of variance (ANOVA) or *t*-test. For muscle performance data, two-way ANOVA was conducted followed by two-tailed Student’s *t*-test. Statistical significances were defined as *p* value less than 0.05.

## Results

### In skeletal muscle, UCHL1 is primarily expressed in oxidative muscle fibers

**Fig 1:**
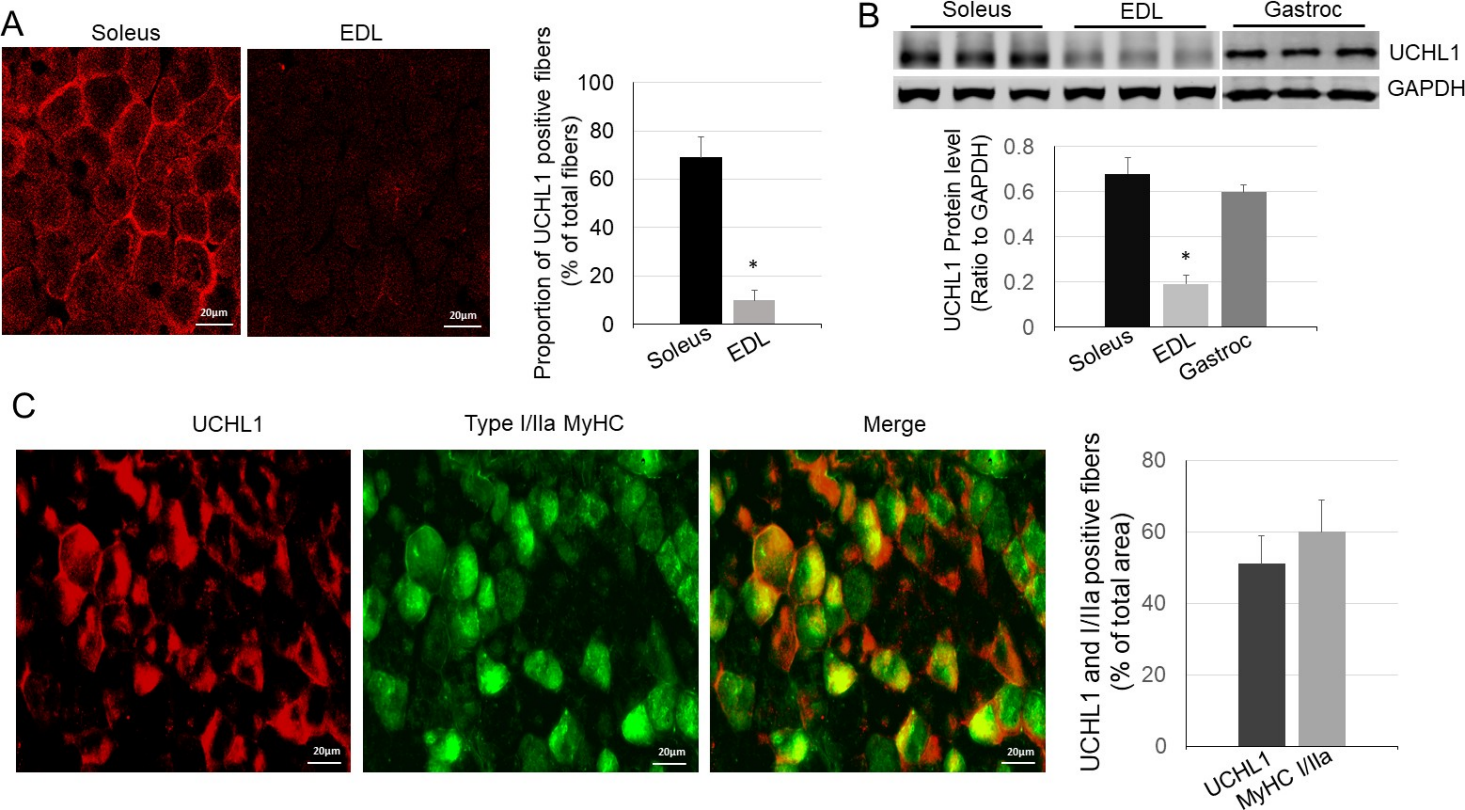
UCHL1 in skeletal muscle is selectively expressed in oxidative muscle fibers. **A:** Representative images and quantification of UCHL1 immunofluorescence of soleus (left) and EDL (right); **B:** Image and quantification of Western blots for UCHL1 and GAPDH from soleus, EDL, and gastrocnemius. The Western blot image was directly copied from the original membrane scan and each lane was as originally loaded without regrouping. * P< 0.05, n = 3; **C:** Images and quantification of immunofluorescence of gastrocnemius muscle with UCHL1 (red) and type I/IIa MyHC (green).

We first compared protein levels of UCHL1 in soleus, a typical slow oxidative muscle, and extensor digitorum longus (EDL), a typical glycolytic fast twitch muscle, using Western blot and immunofluorescent staining. As shown in Fig 1A and 1B, UCHL1 protein was present in soleus but exhibited very low level in EDL. In gastrocnemius, which contains mixed muscle fibers, UCHL1 was primarily present in oxidative muscle fibers (type I and type IIa) as evidenced by its co-localization within MyHC I and IIa positive fibers (Fig 1C). These data indicate that UCHL1 is selectively expressed in oxidative muscle fibers.

### Skeletal muscle specific UCHL1 gene knockout

**Fig 2:**
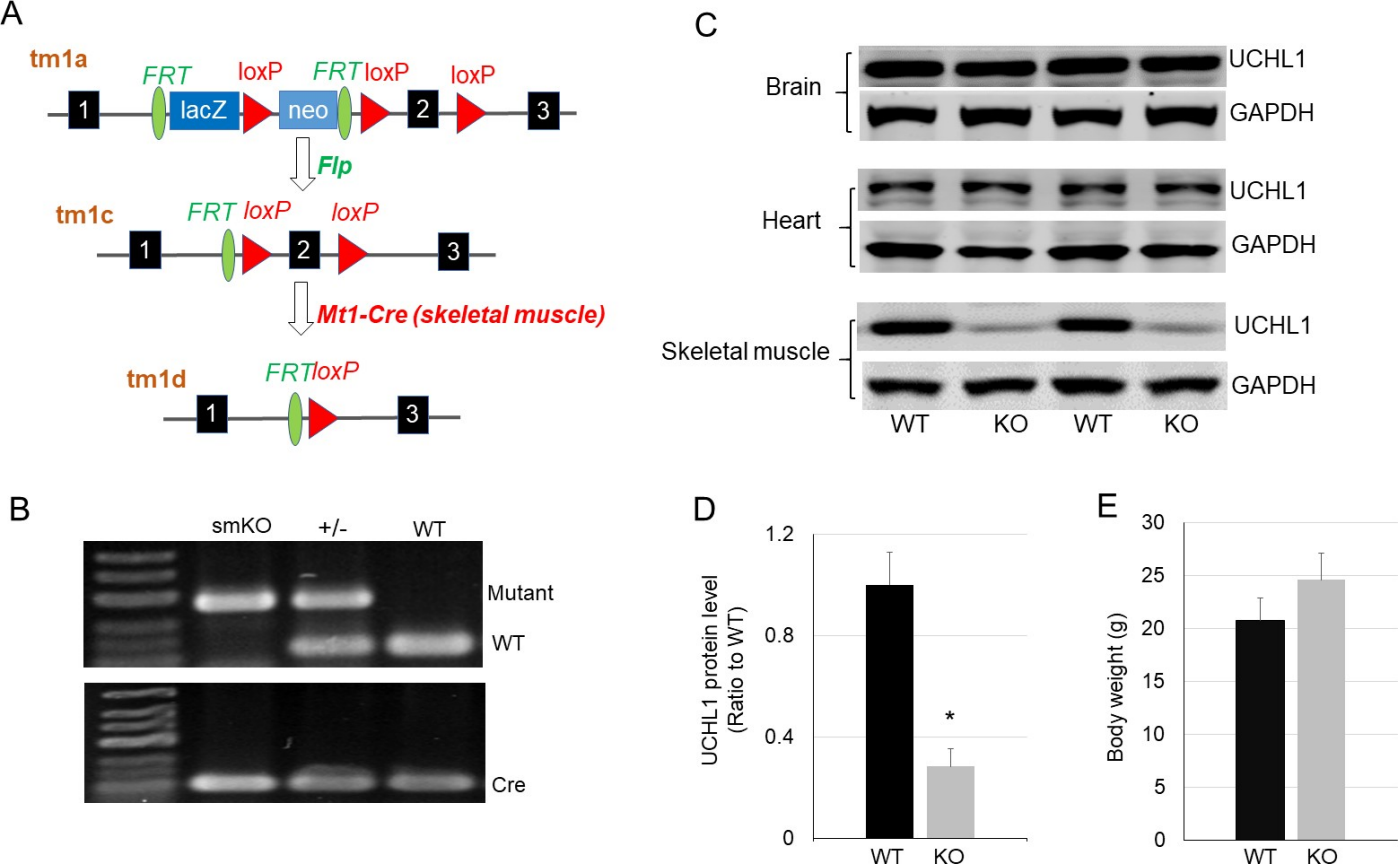
Mouse model of skeletal muscle specific knockout (smKO) of the UCHL1 gene. **A**: Schematic illustration of strategy of UCHL1 smKO. **B**: Representative gel image of PCR genotyping. **C**: Western blot of UCHL1 protein in samples of brain, heart, and skeletal muscles from wild type (WT) or UCHL1 smKO (KO) mice. **D**: Quantification as a ratio of UCHL1 protein band density in WT vs UCHL1 smKO soleus muscle. * P<0.05, n = 4. The representative Western blot images were copied from the original membranes scan and each lane was positioned as originally loaded without regrouping. **E**: Body weight of WT and UCHL1 smKO mice (n = 8).

To understand the function of UCHL1 in skeletal muscle, we generated a mouse model with UCHL1 skeletal muscle specific knockout (UCHL1 smKO, Fig 2A and 2B). Western blot confirmed that UCHL1 expression was specifically reduced in skeletal muscle but not in the brain and heart of smKO mice (Fig 2C and 2D). At 2 months of age, smKO mice showed a trend of increased body weight without statistical significance as compared with WT control (Fig 2E).

### UCHL1 smKO leads to reduced oxidative muscle fibers and oxidative activity

**Fig 3:**
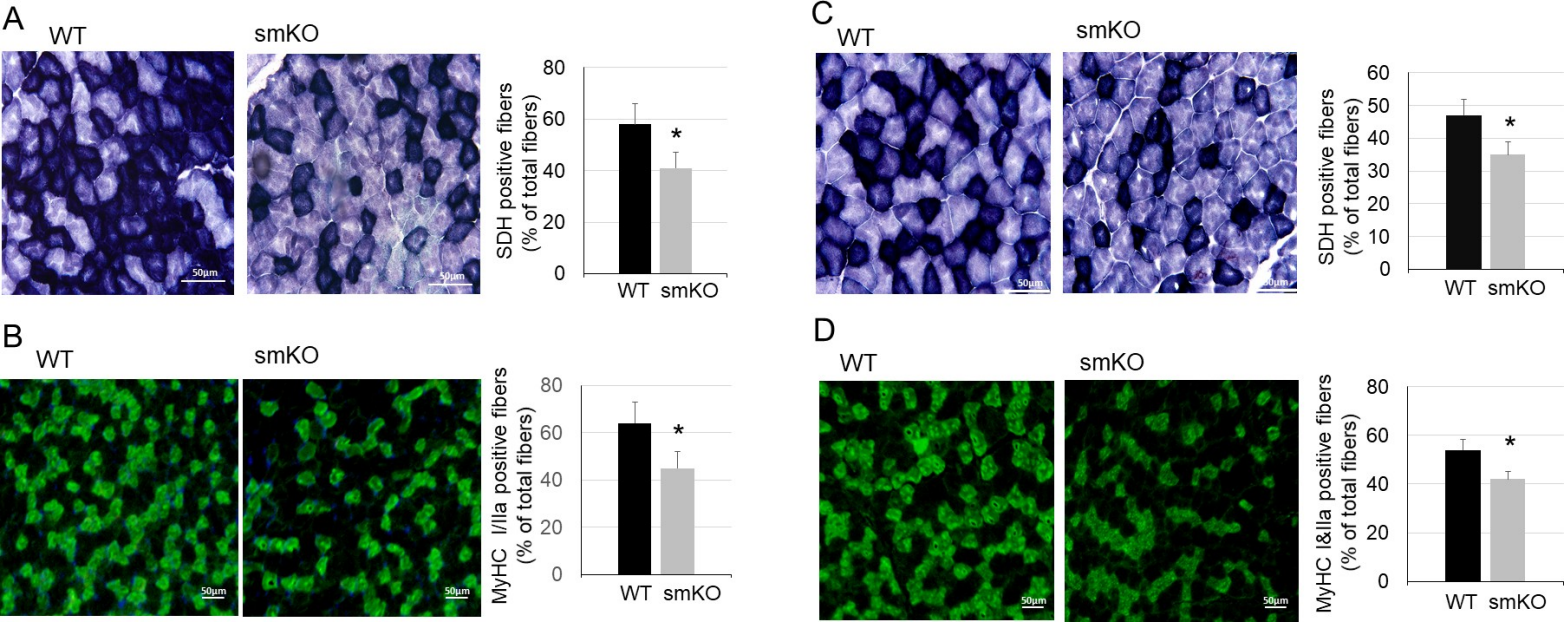
UCHL1 smKO reduces muscle oxidative activity. **A-B**: Representative images and quantifications of SDH staining (A) and type I/IIa MyHC fiber immunofluorescent staining (B) of soleus from WT and UCHL1 smKO mice. C-D: Representative images and quantifications SDH staining (C) and type I/IIa MyHC fiber immunofluorescent staining (D) of gastrocnemius from WT and UCHL1 smKO mice. The quantifications were presented as percentages of positive fibers vs total fibers in a selected area. * P<0.05 vs WT.

Interestingly, in muscles from smKO mice, the oxidative activity measured by succinate dehydrogenase (SDH) staining was significantly reduced as compared with WT mice (Fig 3A and 3C). Consistently, immunostaining showed a significant reduction of oxidative muscle fibers type I/IIa in UCHL1 smKO mice as compared with WT mice (Fig 3B and 3D). These results suggest that UCHL1 may play an important role in maintenance of oxidative metabolism in skeletal muscle.

### UCHL1 deficiency reduces muscle contraction endurance

**Fig 4:**
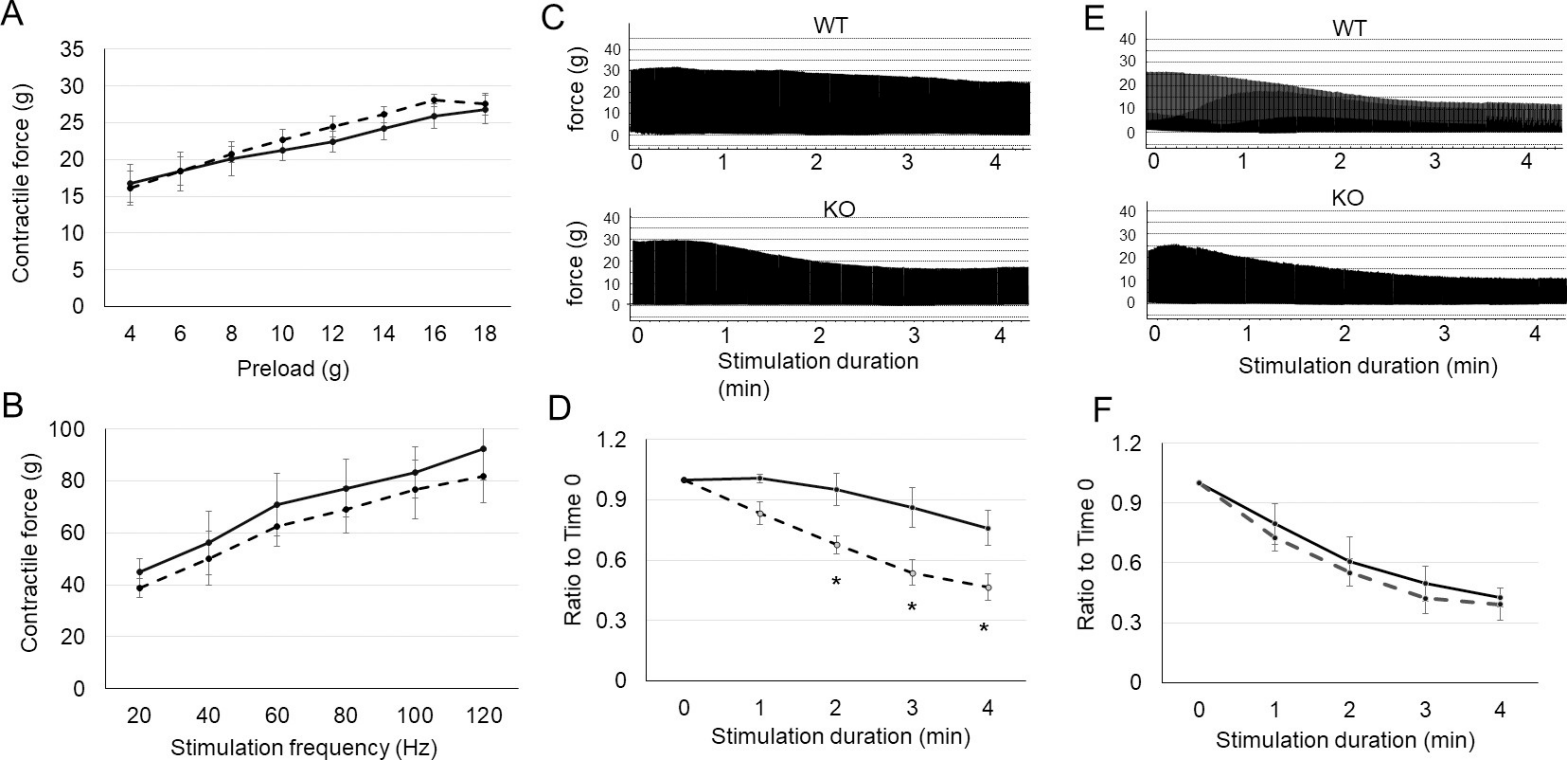
UCHL1 smKO reduces in situ muscle contraction endurance. **A:** Contractile forces of the GPSC from WT (solid line) or UCHL1 (dash line) at various preload**. B:** Frequency-peak contractile force of GPSC from WT (solid curve) or UCHL1 smKO (dash curve); **C-D:** Representative tracings (C) and quantitative data (D) of repetitive single contraction over 4 minutes of GPSC from WT (solid curve) or UCHL1 smKO mice (dash curve). **E-F:** Representative tracings (E) and quantitation (F) of repetitive single contraction over 4 minutes in TA from WT (solid curve) or UCHL1 smKO mice (dash). *P<0.05 vs WT, n = 5.

Oxidative metabolism is critical for skeletal muscle endurance. To test whether the reduction of oxidative activity by UCHL1 smKO may alter skeletal muscle performance, we conducted *in situ* contraction tests of gastrocnemius-plantaris-soleus complex (GPSC) and tibialis anterior (TA) muscles. The maximal contraction force in response to various preload (Fig 4A) and frequency-force response in GPSC (Fig 4B) were not significantly different between UCHL1 smKO and WT muscle. However, the GPSC of UCHL1 smKO mice exhibited faster and greater decline (fatigue) of contractile forces in response to the repetitive stimulation (Fig 4C and 4D). In comparison, the fatigue of TA, a fast muscle, was similar between WT and UCHL1 smKO (Fig 4E and 4F). This data indicates that reduction of oxidative activity by UCHL1 deficiency in muscle results in reduction of muscle endurance.

### UCHL1 deficiency alters key mitochondrial oxidative proteins

**Fig 5:**
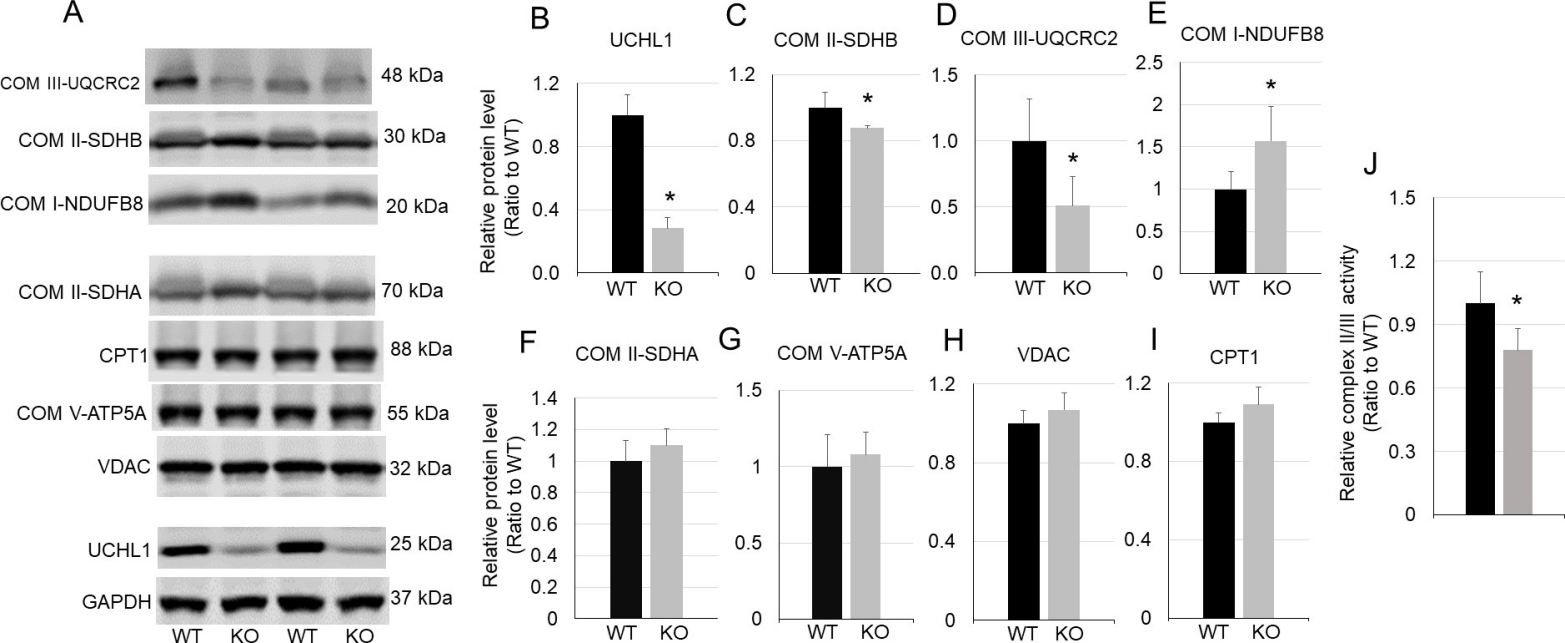
UCHL1 smKO alters mitochondrial OXPHOS proteins. **A-I:** Western blot images (A) and quantifications of Western blot of soleus samples from WT or UCHL1 smKO for UCHL1 (B), SDHB (C), UQCR2 (D), NDUFB8 (E), SDHA (F), ATP5A (G), VDAC (H), and CPT1 (I). **J**: Activity of complex II/III in isolated mitochondria from soleus of WT or UCHL1 smKO. * P<0.05 vs WT, n = 4. The representative Western blot images were copied from the original membranes and each lane was positioned as the originally loaded without regrouping. The original blot images can be found in the S1 File.

Mitochondrion is a critical organelle for oxidative metabolism. We then asked whether UCHL1 smKO affects key proteins that are involved in mitochondrial oxidation in skeletal muscle. Western blot analysis showed that the levels of some proteins of mitochondrial oxidative phosphorylation (OXPHOS) complexes altered in UCHL1 smKO muscles. Specifically, SDHB (subunits of complex II), and UQCRC2 (a component of complex III) were significantly downregulated (Fig 5A, 5C and 5D), whereas NDUFB8, a protein of complex I, was significantly upregulated (Fig 5A and 5E) in soleus with UCHL1 smKO as compared with the control. Other mitochondrial markers including ATP5A (complex V, Fig 5G), VDAC (Fig 5H), and CPT1 (Fig 5I) showed no significant change in UCHL1 smKO soleus as compared with WT muscle. Consistently, the activity of mitochondrial electron transfer chain complex II/III was reduced in soleus from UCHL1 smKO as compared with that from WT (Fig 5J). Moreover, no changes of these OXPHOS proteins were found in EDL muscle (The original blot images can be found in the S1 File), where fast glycolytic fast muscle fibers are predominant and UCHL1 expression is low (Fig 1A and 1B). This data suggests that UCHL1 affects oxidative activity by regulation of mitochondrial OXPHOSE function in oxidative skeletal muscle.

### UCHL1 shows a mitochondrial distribution

**Fig 6:**
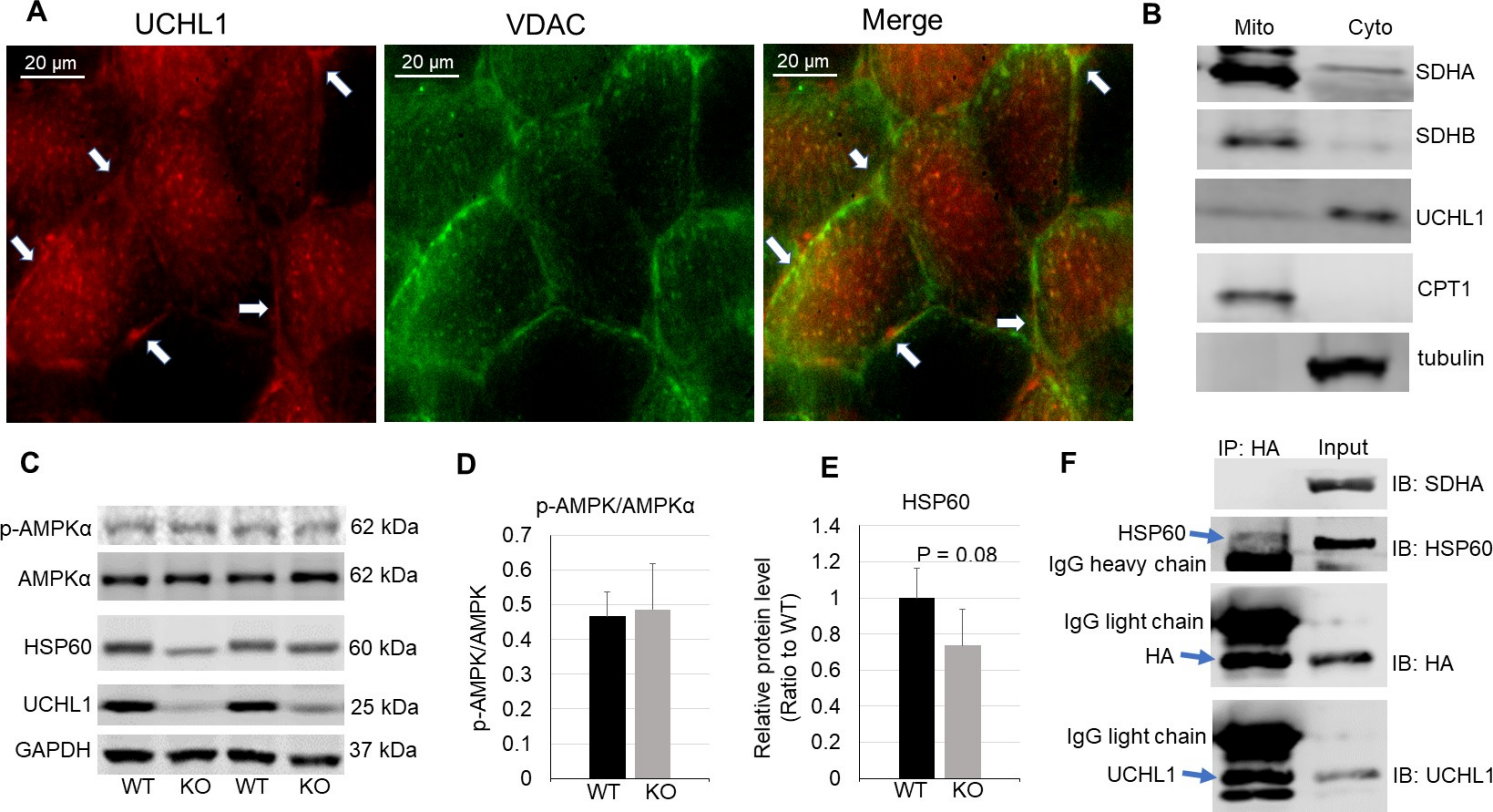
UCHL1 is present in cytosol near mitochondria. **A**: Immunofluorescent staining of UCHL1 (red) and mitochondrial marker VDAC (green) in mouse soleus. The arrows indicate the co-localization of UCHL1 and VDAC staining. **B**: Western blot images of mitochondrial and cytosolic fractionation of gastrocnemius detecting mitochondrial markers SDH and CPT1 in mitochondrial fraction, and UCHL1 and tubulin in cytosolic fraction. **C-E**: Western blot images (C) and quantifications of phospho and total AMPKα (D), and HSP60 (E) in soleus from WT or UCHL1 smKO mice. **F**: Western blot image of HA immunoprecipitation of the protein extract from C2C12 cells with overexpression of HA tagged UCHL1.

We next attempted to further understand how UCHL1 interacts with mitochondrial proteins and regulates mitochondrial function. First, we examined whether UCHL1 was localized within mitochondria. Muscle immunofluorescence staining indicated some co-localization of UCHL1 with the mitochondrial marker VDAC (Fig 6A). To further determine whether UCHL1 is present in mitochondria, we fractionated mitochondria and cytosol from mouse gastrocnemius. Western blot analysis showed that UCHL1 protein was primarily present in the cytosolic fraction. However, there was low level of UCHL1 protein present in mitochondrial fraction (Fig 6B), consistent with the result of immunofluorescent staining. These data suggest that while primarily present in cytosol, UCHL1 may localize near and/or within mitochondria to potentially interact with mitochondrial proteins. The level of phosphorylation of AMPKα, a master regulator of mitochondria biogenesis [25, 26], showed no changes in muscles with UCHL1 smKO as compared with WT muscle (Fig 6C and 6D), suggesting that UCHL1 may not have significant impact on mitochondrial biogenesis. Interestingly, HSP60, a major mitochondrial chaperone protein [27], showed a trend of downregulation in UCHL1 smKO soleus (P = 0.08, Fig 6E). Furthermore, the molecular weight of the HSP60 band seemed lower in UCHL1 smKO muscle, suggesting that UCHL1 may be involved in modification and stabilization of HSP60. To identify proteins that potentially interact with UCHL1, we overexpressed HA-tagged UCHL1 in differentiated C2C12 cells and then conducted HA immunoprecipitation (IP). As seen in Fig 6F, the IP effectively pulled down HA and UCHL1, as well as HSP60, suggesting a direct interaction between UCHL1 and HSP60. On the other hand, no other major mitochondrial proteins, such as SDHA, were detected in the pulldown fraction.

## Discussion

Originally discovered as a neuronal protein, UCHL1 has not been recognized to also express in skeletal muscle until recently [13, 14], and its functional roles in the muscle remain unclear. We recently reported that UCHL1 has an inhibitory effect on myoblast C2C12 differentiation [15]. The results of the present study reveal for the first time that UCHL1 is present in oxidative skeletal muscles and is essential for muscle oxidative activity. This novel information may help us to understand better the function of UCHL1 in skeletal muscle.

Oxidative muscle fibers generate ATP mainly via OXPHOS in mitochondria. OXPHOS involves several processes and many proteins, with five complexes of the electron transport chain being critical. Our data shows that UCHL1 smKO caused a reduction of oxidative activity, measured by muscle SDH staining. Moreover, protein level of SDHB, the component of complex II, and UQCRC2, a component of complex III were significantly reduced in UCHL1 smKO muscle. Consistently, the activity of mitochondrial complex II/III was also reduced in soleus from UCHL1 smKO mice. These data suggest that UCHL1 may play a role in stabilization of these key mitochondrial proteins. On the other hand, UCHL1 smKO did not affect other mitochondrial markers, including ATP5A (complex V), CPT1, and VDAC. Interestingly, the level of NDUFN8, a component of complex I was even upregulated. This may reflect a compensatory response to the reduced complex II activity [28]. Moreover, the phosphorylation of AMPKα, a key regulator of mitochondrial biogenesis, was unchanged in UCHL1 smKO muscle. This suggests that UCHL1 may not play a significant role in overall mitochondrial biogenesis, but rather influence specific OXPHOS proteins.

Ubiquitination and deubiquitination play important roles in regulation of mitochondrial function via the turnover regulation of key mitochondrial proteins [29, 30]. For example, a recent study showed that the degradation of mitochondrial proteins, such as SDHA and SDHB, is regulated by ubiquitination and is proteasome-mediated [30]. As a deubiquitinating enzyme, UCHL1 could deubiquitinate and thus stabilize mitochondrial proteins; therefore, deletion of UCHL1 may increase ubiquitination-induced degradation of OXPHOS proteins such as SDHs. Our immunofluorescence staining showed a co-localization of UCHL1 and a mitochondrial marker VDAC. Moreover, the mitochondrial fractionation assay showed that, while UCHL1 is primarily present in the cytosol, a low level of UCHL1 protein was present in mitochondrial fraction. These data suggest that UCHL1 is localized near and/or even within mitochondria to interact and regulate mitochondrial proteins. However, at this moment, our immunoprecipitation assay was unable to identify a physical interaction of UCHL1 with some major mitochondrial proteins. Further experiments are warranted to determine whether UCHL1 directly interacts with and regulate proteins within mitochondria.

Most of mitochondrial proteins are nuclear-encoded proteins that are translocated into the mitochondria [31]. It is known that some mitochondrial proteins can be ubiquitinated and degraded prior to their import across the mitochondrial membranes [32, 33]. Therefore, it is also possible that cytosolic UCHL1 may regulate mitochondrial proteins such as SDHB and UQCRC2 prior to or during the translocation process of these proteins. The tissue immunofluorescence staining showed a mitochondrial or perimitochondrial localization of UCHL1, further support the possibility of UCHL1 regulation of mitochondrial protein translocation.

Additionally, we also observed that UCHL1 smKO reduces the protein level of HSP60, a key chaperon protein that is critical for mitochondrial protein transport [27, 34]. It is known that HSP60 has dynamic distribution between both cytosol and mitochondria [35]. It is possible that UCHL1 regulates HSP60 turnover and thus indirectly affects mitochondrial translocation of key OXPHOS proteins. This possibility was supported by our data that UCHL1 physically interacts with HSP60 in C2C12 myotubes, and that UCHL1 smKO leads to a trend of downregulation and molecular weight shift of HSP60 in skeletal muscle.

Overall, this study provides novel evidence that UCHL1 is involved in the regulation of oxidative metabolism and function in oxidative skeletal muscle, at least partially through regulation of key OXPHOS proteins and mitochondrial function. Further studies are needed to define and characterize the underlying mechanism by which UCHL1 regulates OXPHOS proteins and mitochondrial function. As the largest tissue mass in the body, skeletal muscle plays an important role in whole body metabolism in physiological conditions such as exercise and aging, and pathological conditions such as obesity, diabetes, and heart failure. It would be interesting and important to determine whether alterations of UCHL1 expression and function are involved in mitochondrial dysfunction in skeletal muscle in disease conditions.

## Supporting information

S1 File (DOCX)
