# Supplementary material for: UCHL1 regulates oxidative activity in skeletal muscle
Source: PLoS One. 2020 Nov 2;15(11):e0241716. doi: 10.1371/journal.pone.0241716 (PMC7605647; doi:10.1371/journal.pone.0241716)
Supplement: S1 File — (DOCX) [file pone.0241716.s001.docx]

|  | 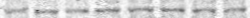  **Original Western blot membrane images**  *The boxes indicate displayed areas as representative images in the figures*  WT KO WT KO WT KO WT KO 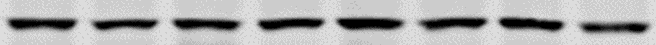 GAPDH  37 kD  UCHL1 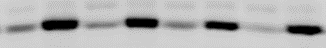 25 kD 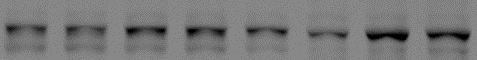 PGC1 alpha  90 kD 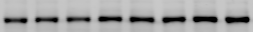 AMPKα  62 kD  HSP60 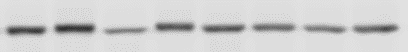 60 kD 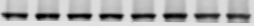 ATP5A  55 kD 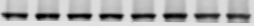 CPT1  88 kD  VDAC 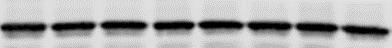 32 kD  NDUFB8 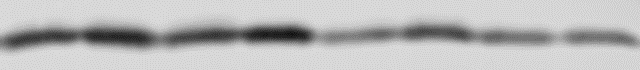 20 kD  UQCRC2  48 kD 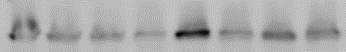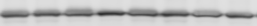 SDHA  70 kD  CII-SDHB  30 kD 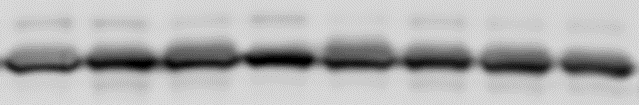 62 kD  pAMPKα   \|  \| \| --- \| |  |  |  |  |  |  |  |  |  |  |
| --- | --- | --- | --- | --- | --- | --- | --- | --- | --- | --- | --- | --- |
|  |  |  |  |  |  |  |  |  |  |  |  |
|  |  |  |  |  |  |  |  |  |  |  |  |
|  |  |  |  |  |  |  |  |  |  |  |  |
|  |  |  |  |  |  |  |  |  |  |  |  |
|  |  |  |  |  |  |  |  |  |  |  |  |
|  |  |  |  |  |  |  |  |  |  |  |  |
|  |  |  |  |  |  |  |  |  |  |  |  |
|  |  |  |  |  |  |  |  |  |  |  |  |
|  |  |  |  |  |  |  |  |  |  |  |  |
|  |  |  |  |  |  |  |  |  |  |  |  |
|  |  |  |  |  |  |  |  |  |  |  |  |
|  |  |  |  |  |  |  |  |  |  |  |  |
|  |  |  |  |  |  |  |  |  |  |  |  |
|  |  |  |  |  |  |  |  |  |  |  |  |
|  |  |  |  |  |  |  |  |  |  |  |  |
|  |  |  |  |  |  |  |  |  |  |  |  |
|  |  |  |  |  |  |  |  |  |  |  |  |
|  |  |  |  |  |  |  |  |  |  |  |  |
|  |  |  |  |  |  |  |  |  |  |  |  |
|  |  |  |  |  |  |  |  |  |  |  |  |
|  |  |  |  |  |  |  |  |  |  |  |  |
|  |  |  |  |  |  |  |  |  |  |  |  |
|  |  |  |  |  |  |  |  |  |  |  |  |
|  |  |  |  |  |  |  |  |  |  |  |  |
|  |  |  |  |  |  |  |  |  |  |  |  |
|  |  |  |  |  |  |  |  |  |  |  |  |
|  |  |  |  |  |  |  |  |  |  |  |  |
|  |  |  |  |  |  |  |  |  |  |  |  |
|  |  |  |  |  |  |  |  |  |  |  |  |
|  |  |  |  |  |  |  |  |  |  |  |  |
|  |  |  |  |  |  |  |  |  |  |  |  |
|  |  |  |  |  |  |  |  |  |  |  |  |
|  |  |  |  |  |  |  |  |  |  |  |  |
|  |  |  |  |  |  |  |  |  |  |  |  |
|  |  |  |  |  |  |  |  |  |  |  |  |
|  |  |  |  |  |  |  |  |  |  |  |  |
|  |  |  |  |  |  |  |  |  |  |  |  |
|  |  |  |  |  |  |  |  |  |  |  |  |
|  |  |  |  |  |  |  |  |  |  |  |  |
|  |  |  |  |  |  |  |  |  |  |  |  |
|  |  |  |  |  |  |  |  |  |  |  |  |
|  |  |  |  |  |  |  |  |  |  |  |  |
|  |  |  |  |  |  |  |  |  |  |  |  |
|  | WT KO WT KO WT KO WT KO |  |  |  |  |  |  |  |  |  |  |
|  |  | | | | | | | |  |  |  |
|  |  |  |  |  |  |  |  |  |  |  |  |
|  |  |  |  |  |  |  |  |  |  |  |  |
| \|  \| \| --- \| |  |  |  |  |  |  |  |  |  |  |  |
|  |  |  |  |  |  |  |  |  |  |  |  |
|  |  |  |  |  |  |  |  |  |  |  |  |
|  |  |  |  |  |  |  | **Quantifications of above Western blot membrane images**  *Final numbers (highlighted columns) are the ratio to WT mean values* |  |  |  |  |
|  |  |  |  |  |  |  |  |  |  |  |  |
|  | Raw |  | ratio to GAPDH | | Ratio to WT Mean | |  |  |  |  |  |
|  | WT | KO | WT | KO | WT | KO | 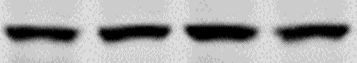 GAPDH |  |  |  |  |
| GAPDH | 14783.32 | 12160.66 |  |  |  |  |  | | |  |  |
|  | 13595.78 | 13733.32 |  |  |  |  |  |  |  |  |  |
|  | 15239.78 | 13732.15 |  |  |  |  |  |  |  |  |  |
|  | 14133.61 | 12834.27 |  |  |  |  | \|  \| \| --- \| |  |  |  |  |
|  |  |  |  |  |  |  |  |  |  |  |  |
| UCHL1 | 13883.2 | 2387.397 | 0.939112 | 0.196321 | 0.918 | 0.192 |  |  |  |  |  |
|  | 13341.66 | 4163.347 | 0.981309 | 0.303157 | 0.959 | 0.296 |  |  |  |  |  |
|  | 14577.9 | 3939.054 | 0.956569 | 0.286849 | 0.935 | 0.280 |  |  |  |  |  |
|  | 17159.51 | 4756.761 | 1.214093 | 0.37063 | 1.187 | 0.362 |  |  |  |  |  |
|  |  | WT mean | 1.022771 |  | 1.000 | 0.283 | mean |  |  |  |  |
|  |  |  |  |  | 0.126 | 0.070 | SD | 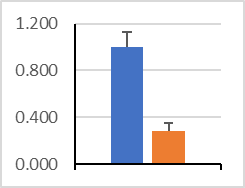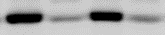 UCHL1 |  |  |  |
|  |  |  |  |  |  | 0.0001 | t test |  |  |  |  |
|  |  |  |  |  |  |  | \|  \| \| --- \| |  |  |  |  |
| SDHB | 14124.58 | 12057.31 | 0.955441 | 0.991501 | 0.864 | 0.897 |  |  |  |  |  |
|  | 16226.57 | 13205.92 | 1.1935 | 0.961598 | 1.079 | 0.870 |  |  |  |  |  |
|  | 17243.28 | 13405.77 | 1.131465 | 0.976233 | 1.023 | 0.883 |  |  |  |  |  |
|  | 16157.82 | 12212.84 | 1.143219 | 0.951581 | 1.034 | 0.860 |  |  |  |  |  |
|  |  | WT mean | 1.105906 |  | 1.000 | 0.877 | mean |  |  |  |  |
|  |  |  |  |  | 0.094 | 0.016 | SD | 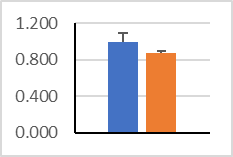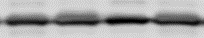 SDHB |  |  |  |
|  |  |  |  |  |  | 0.042 | t |  |  |  |  |
| SDHA | 4878.447 | 4961.255 | 0.329997 | 0.407976 | 1.136 | 1.404 | \|  \| \| --- \| |  |  |  |  |
|  | 4205.447 | 4511.941 | 0.30932 | 0.32854 | 1.065 | 1.131 |  |  |  |  |  |
|  | 4133.447 | 4952.255 | 0.271227 | 0.360632 | 0.933 | 1.241 |  |  |  |  |  |
|  | 3557.841 | 4034.062 | 0.251729 | 0.31432 | 0.866 | 1.082 |  |  |  |  |  |
|  |  | WT mean | 0.290568 |  | 1.000 | 1.214 | mean |  |  |  |  |
|  |  |  |  |  | 0.122 | 0.143 | SD |  |  |  |  |
|  |  |  |  |  |  | 0.063 | t |  |  |  |  |
|  |  |  |  |  |  |  |  | 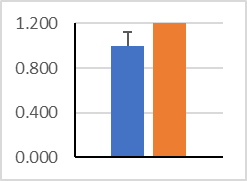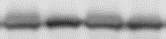 SDHA |  |  |  |
| NDUFB8 | 12060.6 | 17705.92 | 0.815825 | 1.456 | 1.111 | 1.982 | 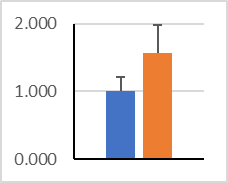 NDUFB8 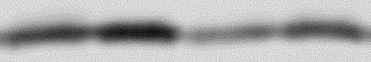  \|  \| \| --- \| |  |  |  |  |
|  | 12281.6 | 17986.65 | 0.903339 | 1.309709 | 1.230 | 1.783 |  |  |  |  |  |
|  | 8820.752 | 14870.95 | 0.578798 | 1.082929 | 0.788 | 1.474 |  |  |  |  |  |
|  | 9048.681 | 9870.731 | 0.640224 | 0.769092 | 0.872 | 1.047 |  |  |  |  |  |
|  |  | WT mean | 0.734547 |  | 1.000 | 1.572 | mean |  |  |  |  |
|  |  |  |  |  | 0.205 | 0.407 | SD |  |  |  |  |
|  |  |  |  |  |  | 0.046 | t |  |  |  |  |
|  |  |  |  |  |  |  |  |  |  |  |  |
|  |  |  |  |  |  |  |  |  |  |  |  |
|  |  |  |  |  |  |  |  |  |  |  |  |
|  |  |  |  |  |  |  |  |  |  |  |  |
|  |  |  |  |  |  |  |  |  |  |  |  |
|  | 13729.58 | 13171.27 | 0.928721 | 1.083105 | 0.992 | 1.157 | 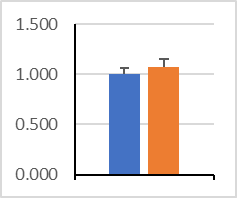 VDAC 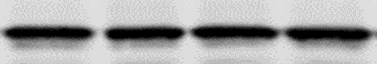  \|  \| \| --- \| |  |  |  |  |
| VDAC   \|  \| \| --- \| | 13746.68 | 12380.97 | 1.011099 | 0.901528 | 1.080 | 0.963 |  |  |  |  |  |
|  | 13144.15 | 13442.39 | 0.862489 | 0.978899 | 0.921 | 1.046 |  |  |  |  |  |
|  | 13316.27 | 13377.92 | 0.94217 | 1.04236 | 1.006 | 1.113 |  |  |  |  |  |
|  |  | WT mean | 0.93612 |  | 1.000 | 1.070 | mean |  |  |  |  |
|  |  |  |  |  | 0.065 | 0.085 | SD |  |  |  |  |
|  |  |  |  |  |  | 0.239 | t |  |  |  |  |
|  |  |  |  |  |  |  |  |  |  |  |  |
| UQCRC2 | 11380.95 | 5665.267 | 0.769851 | 0.465868 | 1.007 | 0.609 | 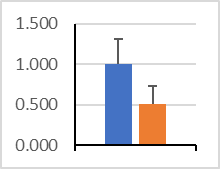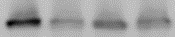 UQCRC2   \|  \| \| --- \| |  |  |  |  |
|  | 6796.752 | 2355.912 | 0.499916 | 0.171547 | 0.654 | 0.224 |  |  |  |  |  |
|  | 16517.16 | 4954.146 | 1.083819 | 0.36077 | 1.418 | 0.472 |  |  |  |  |  |
|  | 9948.551 | 7216.167 | 0.703893 | 0.562258 | 0.921 | 0.736 |  |  |  |  |  |
|  |  | WT mean | 0.76437 |  | 1.000 | 0.510 | mean |  |  |  |  |
|  |  |  |  |  | 0.317 | 0.219 | SD |  |  |  |  |
|  |  |  |  |  |  | 0.044 | t |  |  |  |  |
|  |  |  |  |  |  |  |  |  |  |  |  |
|  |  |  |  |  |  |  |  |  |  |  |  |
|  |  |  |  |  |  |  |  |  |  |  |  |
| ATP5A | 11322.17 | 11903.17 | 0.765875 | 0.978826 | 0.981 | 1.254 | \|  \| \| --- \| |  |  |  |  |
|  | 11240.46 | 10898 | 0.826761 | 0.793544 | 1.059 | 1.017 |  |  |  |  |  |
|  | 11051.65 | 12220.89 | 0.725184 | 0.889948 | 0.929 | 1.140 |  |  |  |  |  |
|  | 11366.89 | 10541.43 | 0.804246 | 0.82135 | 1.030 | 1.052 |  |  |  |  |  |
|  |  | WT mean | 0.780516 |  | 1.000 | 1.116 | mean |  |  |  |  |
|  |  |  |  |  | 0.057 | 0.106 | SD |  |  |  |  |
|  |  |  |  |  |  | 0.102 | t |  |  |  |  |
|  |  |  |  |  |  |  |  |  |  | 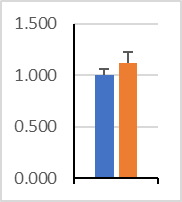 ATP5A 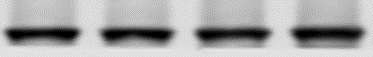 |  |
|  |  |  |  |  |  |  |  |  |  |  |  |
|  |  |  |  |  |  |  |  |  |  |  |  |
| CPT1 | 14936.33 | 15018.21 | 1.01035 | 1.234983 | 0.996 | 1.217 | 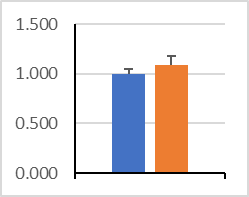 CPT1 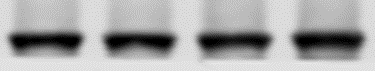  \|  \| \| --- \| |  |  |  |  |
|  | 14632.84 | 14238.45 | 1.076278 | 1.036782 | 1.061 | 1.022 |  |  |  |  |  |
|  | 14660.23 | 15158.82 | 0.961971 | 1.103893 | 0.948 | 1.088 |  |  |  |  |  |
|  | 14264.99 | 13515.82 | 1.009295 | 1.053104 | 0.995 | 1.038 |  |  |  |  |  |
|  |  | WT mean | 1.014474 |  | 1.000 | 1.091 | mean |  |  |  |  |
|  |  |  |  |  | 0.046 | 0.089 | SD |  |  |  |  |
|  |  |  |  |  |  | 0.117 | t |  |  |  |  |
|  |  |  |  |  |  |  |  |  |  |  |  |
|  |  |  |  |  |  |  |  |  |  |  |  |
|  |  |  |  |  |  |  |  |  |  |  |  |
| HSP60 | 8951.296 | 5020.811 | 0.6055 | 0.412873 | 0.960 | 0.654 | 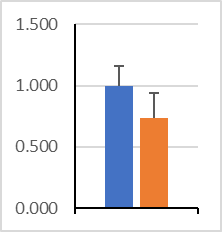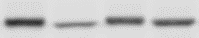 HSP60   \|  \| \| --- \| |  |  |  |  |
|  | 7355.882 | 7416.054 | 0.541041 | 0.540005 | 0.858 | 0.856 |  |  |  |  |  |
|  | 9121.539 | 4301.861 | 0.598535 | 0.313269 | 0.949 | 0.497 |  |  |  |  |  |
|  | 11004.2 | 7624.882 | 0.778584 | 0.594103 | 1.234 | 0.942 |  |  |  |  |  |
|  |  | WT mean | 0.630915 |  | 1.000 | 0.737 | mean |  |  |  |  |
|  |  |  |  |  | 0.163 | 0.201 | SD |  |  |  |  |
|  |  |  |  |  |  | 0.088 | t |  |  |  |  |
|  |  |  |  |  |  |  |  |  |  |  |  |
|  |  |  |  |  |  |  |  |  |  |  |  |
|  | p-AMPK |  | total AMPK | | p-AMPK/AMPK | |  |  |  |  |  |
|  | WT | KO | WT | KO | WT | KO |  |  |  |  |  |
|  | 3147.125 | 3576.518 | 6395.347 | 6923.296 | 0.492096 | 0.516592 | pAMPKα  AMPKα 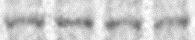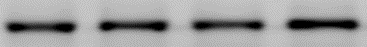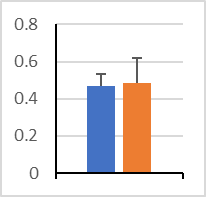  \|  \| \| --- \| |  |  |  |  |
|  | 3221.761 | 4003.317 | 7461.933 | 6078.104 | 0.43176 | 0.658646 |  |  |  |  |  |
|  | 4467.439 | 3024.853 | 8123.296 | 7868.175 | 0.549954 | 0.384442 |  |  |  |  |  |
|  | 3651.246 | 3461.69 | 9250.175 | 9038.882 | 0.394722 | 0.382978 |  |  |  |  |  |
|  |  |  |  |  | 0.467133 | 0.485664 | mean |  |  |  |  |
|  |  |  |  |  | 0.068257 | 0.131237 | SD |  |  |  |  |
|  |  |  |  |  |  | 0.810523 | t |  |  |  |  |
|  |  |  |  |  |  |  |  |  |  |  |  |
|  |  |  |  |  |  |  |  |  |  |  |  |
|  |  |  |  |  |  |  |  |  |  |  |  |
|  |  |  |  |  |  |  |  |  |  |  |  |
|  |  |  |  |  |  |  | \|  \| \| --- \| |  |  |  |  |
| PGC1 | 6264.388 | 5487.681 | 0.423747 | 0.451265 | 0.748 | 0.796 |  |  |  |  |  |
|  | 8325.924 | 7708.56 | 0.61239 | 0.561304 | 1.081 | 0.991 |  |  |  |  |  |
|  | 6388.146 | 4692.004 | 0.419176 | 0.34168 | 0.740 | 0.603 |  |  |  |  |  |
|  | 11466.51 | 9981.874 | 0.811294 | 0.777752 | 1.432 | 1.373 |  |  |  |  |  |
|  |  | WT mean | 0.566652 |  | 1.000 | 0.941 | mean |  |  |  |  |
|  |  |  |  |  | 0.329 | 0.329 | SD |  |  |  |  |
|  |  |  |  |  |  | 0.807 | t |  |  |  |  |
|  |  |  |  |  |  |  |  |  |  |  |  |
|  |  |  |  |  |  |  | 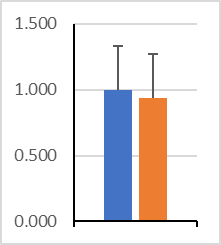 PGC1 alpha 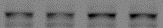 |  |  |  |  |
|  |  |  |  |  |  |  |  |  |  |  |  |
|  |  |  |  |  |  |  |  |  |  |  |  |
|  |  |  |  |  |  |  |  |  |  |  |  |
|  |  |  |  |  |  |  |  |  |  |  |  |
|  |  |  |  |  |  |  |  |  |  |  |  |
|  |  |  |  |  |  |  |  |  |  |  |  |
|  |  |  |  |  |  |  |  |  |  |  |  |
|  |  |  |  |  |  |  |  |  |  |  |  |
|  |  |  |  |  |  |  |  |  |  |  |  |
|  |  | | | | | | | | |  |  |
|  |  |  |  |  |  |  |  |  |  |  |  |
| Western blot with EDL muscle samples |  |  |  |  |  |  |  |  |  |  |  |
|  |  |  |  |  |  |  |  |  |  |  |  |
| 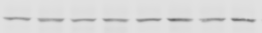 HSP60- 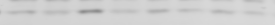 UCHL1  KO WT KO WT KO WT KO WT 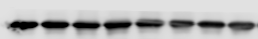 GADPH  SDHA 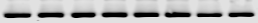 NDUFB8- 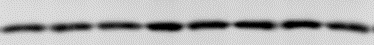 UQCRC2 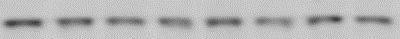 SDHB 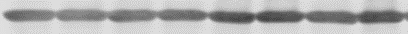 25 kD  60 kD  30 kD  70 kD  20 kD  48 kD  37 kD |  |  |  |  |  |  |  |  |  |  |  |
|  |  |  |  |  |  |  |  |  |  |  |  |
|  |  |  |  |  |  |  |  |  |  |  |  |
|  |  |  |  |  |  |  |  |  |  |  |  |
|  |  |  |  |  |  |  |  |  |  |  |  |
|  |  |  |  |  |  |  |  |  |  |  |  |
|  |  |  |  |  |  |  |  |  |  |  |  |
|  |  |  |  |  |  |  |  |  |  |  |  |
|  |  |  |  |  |  |  |  |  |  |  |  |
|  |  |  |  |  |  |  |  |  |  |  |  |
|  |  |  |  |  |  |  |  |  |  |  |  |
|  |  |  |  |  |  |  |  |  |  |  |  |
|  |  |  |  |  |  |  |  |  |  |  |  |
|  |  |  |  |  |  |  |  |  |  |  |  |
|  |  |  |  |  |  |  |  |  |  |  |  |
|  |  |  |  |  |  |  |  |  |  |  |  |
|  |  |  |  |  |  |  |  |  |  |  |  |
|  |  |  |  |  |  |  |  |  |  |  |  |
|  |  |  |  |  |  |  |  |  |  |  |  |
|  |  |  |  |  |  |  |  |  |  |  |  |
|  |  |  |  |  |  |  |  |  |  |  |  |
|  |  |  |  |  |  |  |  |  |  |  |  |
|  |  |  |  |  |  |  |  |  |  |  |  |
